# The American Dental Association

**Published:** 1871-08

**Authors:** 


### EDITORIAL DEPARTMENT.

*The American Dental Association.*—Dr. Cushing sends us the following information, which has been received from the various members of the committee to secure reduced rates of travel for delegates to the American Dental Association.

*New Orleans:* All roads from that city will carry at half fare.

*Louisville:* Half fare.

*Cincinnati:* Half fare.

*St. Louis:* Not heard from.

*Chicago:* No arrangement has been yet completed; if any is made, due notice will be published in the daily and weekly press.

*New York:* Fare for round trip, $21.60. Tickets to be had at 111 Liberty Street, New York City.

*Boston:* All delegates from Boston or New England, will have to go *via* New York. Tickets for round trip from Boston can be obtained at 82 Washington Street, Boston, at $31.60. or through tickets from New York as above.

*San Francisco:* Half fare has been secured over the road from Omaha to Chicago, and it is expected that the same will be secured over the entire road.

Members who cannot avail themselves of half-fare tickets from any of the above central points, or who can only get reduced rates to Baltimore or Washington, will find tickets from Washington to White Sulphur Springs and return, at 603 Pennsylvania Avenue.

*Washington:* Fare for round trip, $12.50.

The promptness and liberality with which the agents of the Orange, Alexandria and Mannasses, and the Ohio and Chesapeake Roads, responded to the request of the committee, demands notice at our hands, as being in pleasing contrast to the action of some of the roads, who grant very reluctantly, if at all, the desired concession.

